# Diversity Patterns of Eukaryotic Phytoplankton in the Medog Section of the Yarlung Zangbo River

**DOI:** 10.1007/s00248-024-02371-6

**Published:** 2024-04-15

**Authors:** Huan Zhu, Shuyin Li, Zhihua Wu, Xiong Xiong, Pengcheng Lin, Benwen Liu, Dekui He, Guoxiang Liu

**Affiliations:** 1 Institute of Hydrobiology, Chinese Academy of Sciences, Wuhan, 430072 China; 2 Eco-Environmental Monitoring and Scientific Research Center, Yangtze River Basin Ecological Environment Supervision and Management Bureau, Ministry of Ecology and Environment, Wuhan, 430010 China; 3 College of Science, Tibet University, Lhasa, 850000 China

**Keywords:** Biodiversity, Southern Tibet, Global changes, Climate warming and humidification, Driving factors

## Abstract

As one of the important biodiversity conservation areas in China, the ecosystem in the lower reaches of the Yarlung Zangbo River is fragile, and is particularly sensitive to global changes. To reveal the diversity pattern of phytoplankton, the metabarcode sequencing was employed in the Medog section of the lower reaches of the Yarlung Zangbo River during autumn 2019 in present study. The phytoplankton assemblies can be significantly divided into the main stem and the tributaries; there are significant differences in the phytoplankton biomass, alpha and beta diversity between the main stem and the tributaries. While both the main stem and the tributaries are affected by dispersal limitation, the phytoplankton assemblages in the entire lower reaches are primarily influenced by heterogeneous selection. Community dissimilarity and assembly process were significantly correlated with turbidity, electrical conductivity, and nitrogen nutrition. The tributaries were the main source of the increase in phytoplankton diversity in the lower reaches of the Yarlung Zangbo River. Such diversity pattern of phytoplankton in the lower reach may be caused by the special habitat in Medog, that is, the excessive flow velocity, and the significant spatial heterogeneity in physical and chemical factors between stem and tributaries. Based on the results and conclusions obtained in present study, continuous long-term monitoring is essential to assess and quantify the impact of global changes on phytoplankton.

**Supplementary Information:**

The online version contains supplementary material available at 10.1007/s00248-024-02371-6.

## Introduction

Phytoplankton plays a crucial role as primary producers in aquatic ecosystems, influencing energy flow and biogeochemical cycles in water ecosystems. The community diversity of phytoplankton is closely related to the physicochemical environment and hydrological conditions of water bodies, providing a visual reflection of the impact of climate change or human activities on aquatic ecosystems [36]. The formation and driving factors of phytoplankton diversity patterns are generally considered to be determined by four processes similar to other types of microorganisms: speciation, selection, drift, and dispersal [30]. These processes interact and collectively shape the global or regional patterns of phytoplankton on different temporal and spatial scales. Most classical theoretical models, experimental simulations, and field studies suggest that in river ecosystems, the diversity patterns of phytoplankton are primarily influenced by the processes of dispersal and selection [25]. The relative strength of dispersal and selection processes in phytoplankton assemblies in different flow rate water bodies often changes during different stages of community succession. In fast-flowing rivers, dispersal limitation usually dominates the early stages of community succession. However, in later stages, both selection and dispersal limitation processes play significant roles [2]. Furthermore, spatial scale is also one of the factors influencing community and community assembly processes [5].

The Yarlung Zangbo River, China’s largest river on the Tibetan Plateau, meanders over 2200 km, exhibiting diverse ecosystems across its upper, middle, and lower reaches. Characterized by wide plateaus and a high plateau semi-arid climate, the upper and middle reaches contrast with the lower reaches, forming a canyon river with swift currents and a tropical rainforest climate. Medog, located in the lower reaches, contributes to the unique biodiversity of the Yarlung Zangbo. Tributaries like Jinzhu Zangbo and Yanglang Zangbo originate from glaciers, shaping a distinctive river landscape from icy zones to subtropical and tropical zones. This geological and climatic diversity fosters a unique biodiversity pattern and a distinct aquatic ecosystem in the Medog (Yang 1983). Due to the variation in important environmental factors such as water, heat, and light, altitude gradient has become a significant aspect in the study of biodiversity patterns [12]. Thus, the Medog section of the Yarlung Zangbo River exhibits a substantial altitude drop over a short distance, rendering its ecosystems highly sensitive to human activities and climate change [7, 28]. While biodiversity studies in the downstream region mainly focus on terrestrial ecosystems, aquatic organisms, particularly phytoplankton, remain understudied [18, 20, 21, 39]. Addressing this research gap is essential for a comprehensive understanding of the Yarlung Zangbo’s fragile and dynamic aquatic ecosystems.

In recent years, the impact of global changes on plateau ecosystems has become increasingly evident. Influenced by the warming and humidifying climate on the plateau, the upstream inflow of the Yarlung Zangbo River has significantly increased. Simultaneously, human activities such as road construction along the river and hydropower development have led to noticeable alterations in the hydrological conditions of certain sections of the main stem and tributaries of the Yarlung Zangbo [37]. Against this backdrop, understanding the baseline phytoplankton diversity of the main stem and tributaries in the Medog segment of the Yarlung Zangbo is fundamental for regional aquatic biological resource conservation. Additionally, all main stems and tributaries of the Yarlung Zangbo within Medog are characterized by canyon river flow, where high water velocities impede the upstream dispersion of numerous aquatic organisms. This flow pattern also restricts the exchange of aquatic biological assemblies among different tributaries, and the horizontal movement between the main stem and tributaries may primarily occur in a unidirectional manner. Therefore, analyzing and characterizing the eukaryotic phytoplankton diversity patterns in this typical aquatic habitat is of significant theoretical importance for understanding the mechanisms governing the formation and maintenance of eukaryotic phytoplankton community diversity.

In recent years, the Uthermoll sedimentation method and microscopic observation for algae identification have encountered numerous challenges due to the phenotypic plasticity and cryptic diversity of algae [40]. Metabarcoding sequencing offers an improved alternative to overcome these limitations associated with traditional microscopic identification techniques (Blaxter, 2004). In this study, high-throughput DNA barcoding sequencing technology was employed to conduct a systematic analysis of the phytoplankton assemblies in the main stem and major tributaries of the Yarlung Zangbo in the Medog segment. The aim was to reveal the distribution patterns of phytoplankton in the Yarlung Zangbo Medog segment, identify the primary influencing factors, and understand the mechanisms sustaining these patterns. This research provides a scientific basis for the rational conservation and sustainable development of aquatic biological resources in the downstream Yarlung Zangbo.

## Materials and Methods

### Phytoplankton Sampling and Evaluation of Environmental Variables

**Fig. 1:**
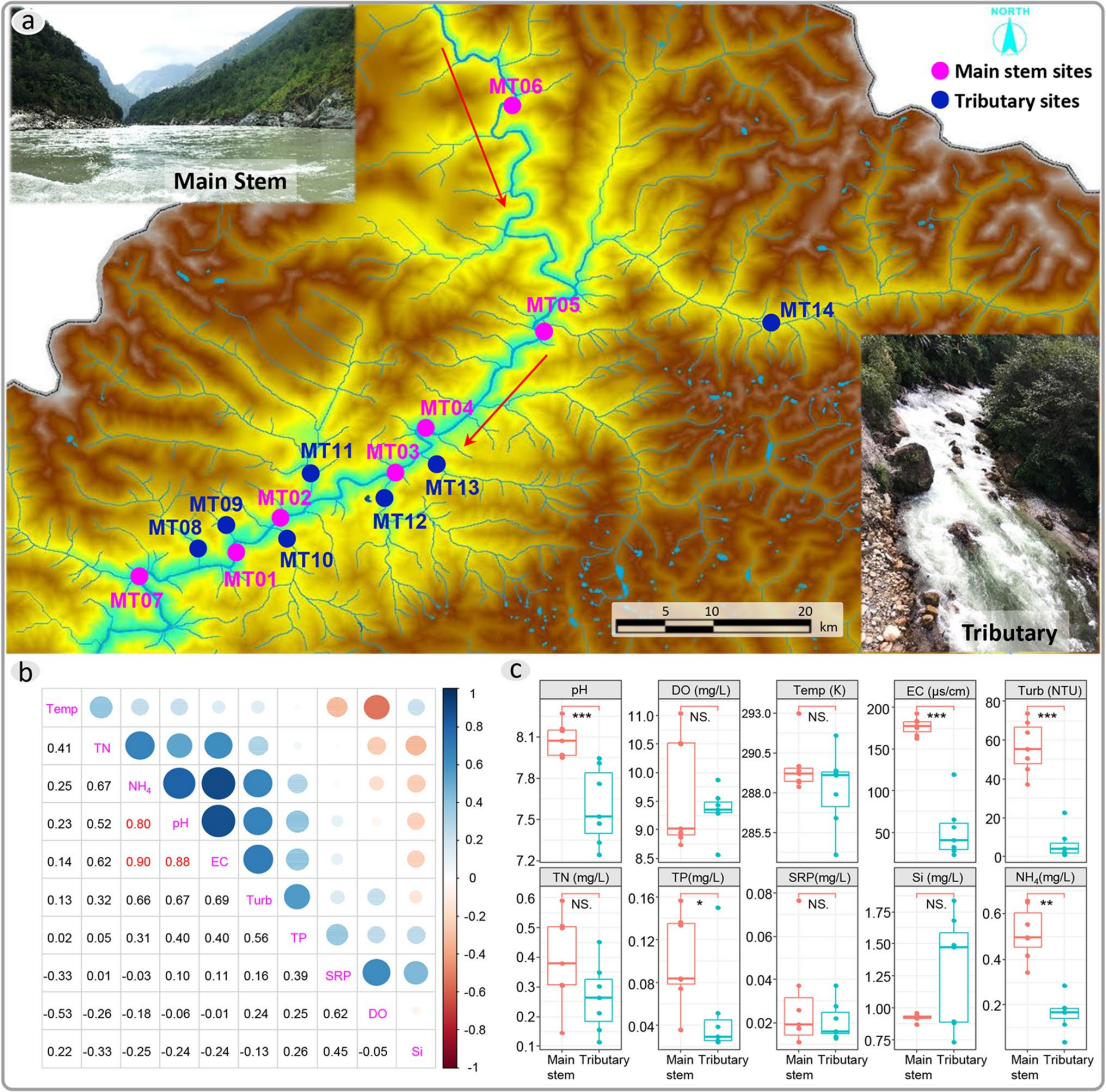
**a** Site distribution in the Medog segment of the Yarlung Zangbo river, showing habitat images of typical main stem and tributaries. Pink dots and blue dots represent main stem sampling sites and tributary sampling sites respectively, the red arrows show the flow direction; **b** Spearman correlation analysis of physicochemical index and nutrient concentrations in the downstream of the Yarlung Zangbo river, the size and color of circle represent correlation ship; **c** Comparison of various physicochemical index and nutrient concentrations between the main stem and tributaries in the Medog segment of the Yarlung Zangbo river, the *, **, and *** represent *p* values < 0.05, < 0.01, and < 0.001, respectively

To represent diverse hydrological dynamics and urban development conditions along the downstream Yarlung Zangbo River and to achieve a balance between water characteristics and road accessibility, a total of 14 sampling sites were selected during 14–27 October 2019. Seven points were designated along the main stem, with an additional seven points along various tributaries (Fig. 1a). All water samples were pre-sieved using a plankton net with a pore diameter of approximately 180 μm to remove large planktonic animals and suspended solids. Three liters of water were collected at each site and then transported to the laboratory. Environmental DNA samples were enriched using a 0.22-μm polycarbonate filter (Millipore) with a 1-l water. Chlorophyll-a concentration (Chla) was used to estimate phytoplankton biomass, measured by filtering 1 l of water through a GF/C membrane (with a diameter 0.45 μm, Waterman). All filters were stored in liquid nitrogen for further analysis. The OMEGA water DNA kit (D5525-01, OMEGA biotech) was used for DNA extraction. An ultra-micro spectrophotometer (Nanodrop 8000, Thermo) was used to test the quality of extracted total DNA. The universal eukaryotic V4 SSU rRNA region primers (forward primer CCAGCASCYGCGGTAATTCC, reverse primer ACTTTCGTTCTTGATYRA) were used [29]. The constructed libraries were tested by Qsep-400 and then sequenced on Hiseq 2500 (Illumina). All raw data were uploaded to the Sequence Read Archive (SRA, National Center for Biotechnology Information).

A portable multi-parameter water quality analyzer (Hydrolab, Hash) was used in filed for measuring physicochemical index like water temperature (Kelvin temperature), dissolved oxygen (DO), pH, and electronical conductivity (EC). Turbidity (Turb) was measured with a portable turbidity meter (2100Q, Hash). Other environmental factors such as chlorophyll-a concentration (Chla), total nitrogen (TN), ammonium nitrogen (NH_4_-N), total phosphorus (TP), and soluble reactive phosphorus (SRP) were determined following the “Methods for the Monitoring and Analysis of Water and Wastewater” (Fourth Edition) [35]. The variance inflation factor (VIF) in regression analysis was used to detect the multicollinearity across environmental variables, and variables with VIF values greater than 10 were deleted in following analysis.

### Sequencing and Annotation of Metabarcoding Data, Statistical Analysis

The raw data from meta-barcoding sequencing were primarily processed using Usearch (v11.0.667) for filtering, quality control, primer trimming, merging, chimera removal, and OTU clustering [11]. Sequences with a similarity of ≥ 97% were grouped into operational taxonomic units (OTUs) using USEARCH (v10.0). OTUs with abundances lower than 0.005% were filtered out, and chimera removal was performed using UCHIME (version 8.1) [10]. The representative sequences of each OTU were annotated with USEARCH (v10.0), and only OTUs corresponding to eukaryotic algae were further analyzed [9]. Their community matrix was rarefied based on the sample with the lowest sequencing depth. The resulting sequence matrix was aligned with MAFFT, trimmed using TrimAl, and used to construct a maximum likelihood tree with IQtree [23]. The majority consensus tree was then employed for calculating alpha and beta phylogenetic diversity, and analyzing community assembly processes. The community matrix was first Hellinger transformed, and environmental variables matrix (except pH) was log(x + 1) transformed using VEGAN package. Alpha diversity (Shanno-Wiener, and phylogenetic diversity indices based on whole tree phylogenetic distance) and principal coordinate analysis (PCoA) were performed using Vegan and PICANTE packages [17, 24]. Beta diversity (Sorensen dissimilarity and phylogenetic Sorensen dissimilarity) was calculated and further divided into species replacement and richness difference components based on both taxonomic and phylogenetic β-diversity using betapart and adespatial packages [1, 8]. βNTI and RCbray values were calculated using picante package, and assembly processes were assessed and quantified with iCAMP package (Stegen et al., 2013). The contribution percentage of homogeneous selection (HoS) was determined based on the percentage of pairwise comparisons with βNRI values less than − 1.96, while heterogeneous selection (HeS) was determined based on those with βNRI values greater than 1.96. Additionally, the contribution percentage of homogenizing dispersal (HD) was calculated from the percentage of pairwise comparisons with |βNRI|≤ 1.96 and RC <  − 0.95, while dispersal limitation (DL) was determined from those with|βNRI|≤ 1.96 and RC > 0.95. Ecological drift (ED) was assessed based on the percentage of pairwise comparisons with |βNRI|≤ 1.96 and |RC|≤ 0.95 [22]. Sink-source analysis of phytoplankton community composition along the main stem was traced with the FEAST package [27]. In the sink-source analysis, we focused solely on downstream points located between adjacent main stem sampling sites where investigated tributaries merged during this survey,therefore, points MT06 and MT04 were excluded (Fig. 1a). Correlations among environmental factors, spatial distance, beta diversity, and community assembly processes (βNTI) were explored using the mantel test in VEGAN package. All analyses were conducted in R.

## Results

### Water Environmental Variables

Spearman correlation analysis revealed a significant correlation (*r* > 0.8) among pH, EC, and NO^3^-N in the Medog segment of the Yarlung Zangbo River. Strong correlations were also observed between TN and NH_4_-N, SRP and DO, as well as TURB and pH, EC, and NO^3^-N (Fig. 1b). In comparing environmental factors between the main stem and tributaries, significant differences were found in pH, EC, Turb, NH_4_-N (*p* < 0.001), and TP (*p* < 0.05) (Fig. 1c). Subsequently, a variance inflation factor (VIF) analysis was conducted, and pH and NH_4_-N were removed from the subsequent analysis based on VIF values below 10.

### Alpha Diversity and Biomass Distribution

**Fig. 2:**
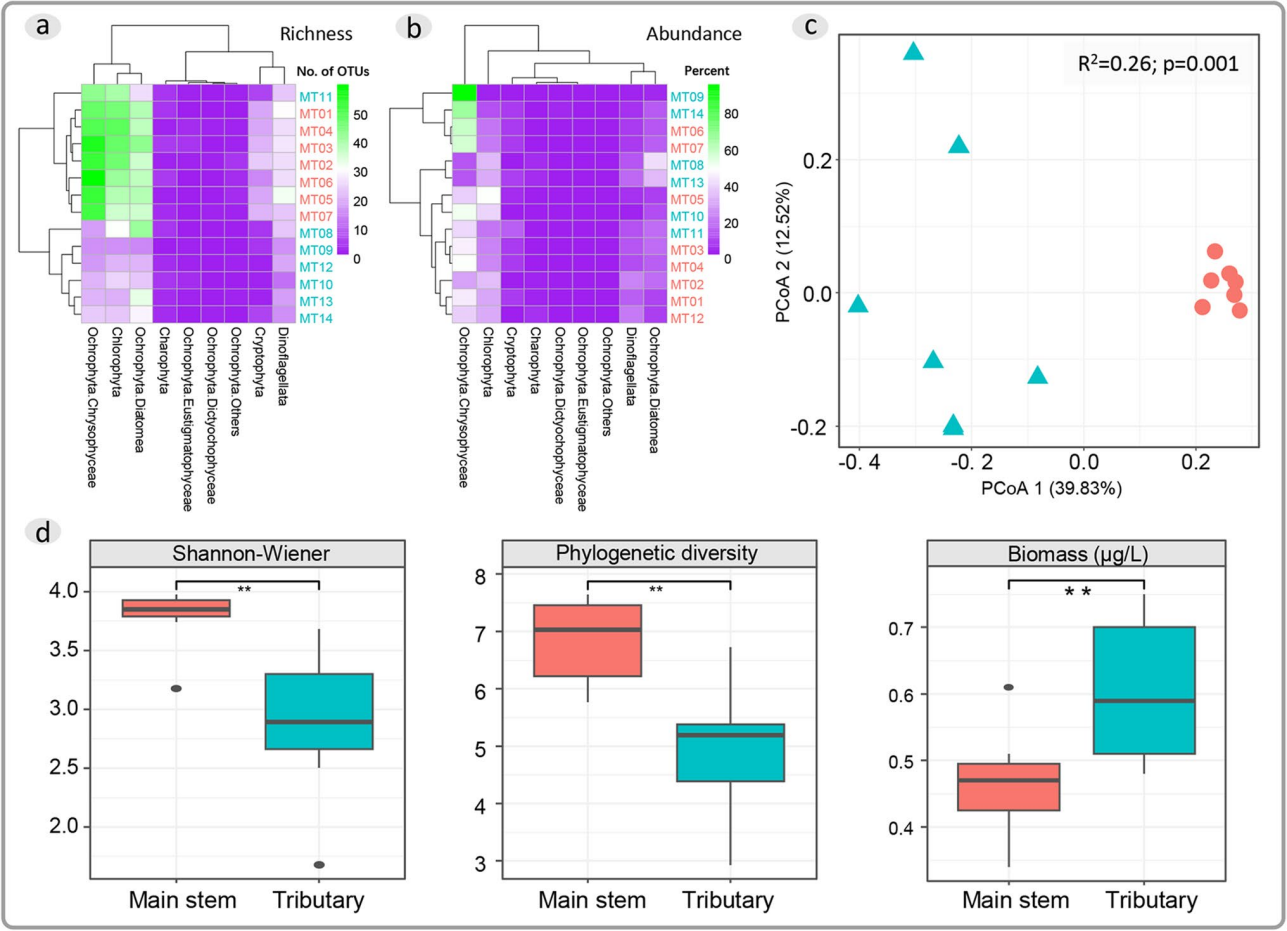
**a** Number of detected OTUs at each sampling site; **b** relative abundance of each algal group at each sampling site; **c** PCoA of 14 assemblies based on Bray–Curtis Distances, with adonis analysis *r*^2^ = 0.26 and *p* = 0.001; **d** comparison of taxonomic alpha diversity, phylogenetic alpha diversity, and biomass (Chla concentration) between the main stem and tributaries. Red words or red circles represent main stem sampling sites, while blue words or blue triangles represent sampling sites in tributaries. The *, **, and *** represent *p* values < 0.05, < 0.01, and < 0.001, respectively

A total of 564 eukaryotic algae OTUs from Chlorophyta, Charophyta, Cryptophyta, Dinoflagellata, and Ochrophyta were identified. Further detailed classification was conducted due to the high richness and abundance observed in Ochrophyta, including taxa such as Chrysophyceae, Eustigmatophyceae, Dictyochophyceae, Diatomea, and others. Both main stem and tributaries showed high richness in Chrysophyceae, Chlorophyta, and Diatomea, followed by Dinoflagellata (Fig. 2a). The main stem generally exhibited significantly higher richness in Chrysophyceae, Chlorophyta, Diatomea, Dinoflagellata, and Cryptophyta than tributaries (Fig. 2a). Most sites were dominated by Chrysophyceae and Chlorophyta, with some exceeding 60% relative abundance (Fig. 2b). Principal coordinates analysis (PCoA) based on Bray–Curtis distances revealed a significant difference between main stem and tributaries (*R*^2^ = 0.26, *p* = 0.001), with higher dissimilarity among tributaries (Fig. 2c). There is significant differences alpha diversity between the main stem and tributaries. Both Shannon–Wiener and phylogenetic diversity indices showed higher in main stem sites. In contrast, the biomass showed significantly higher in the tributaries than main stem (Fig. 2d).

### Beta Diversity

**Fig. 3:**
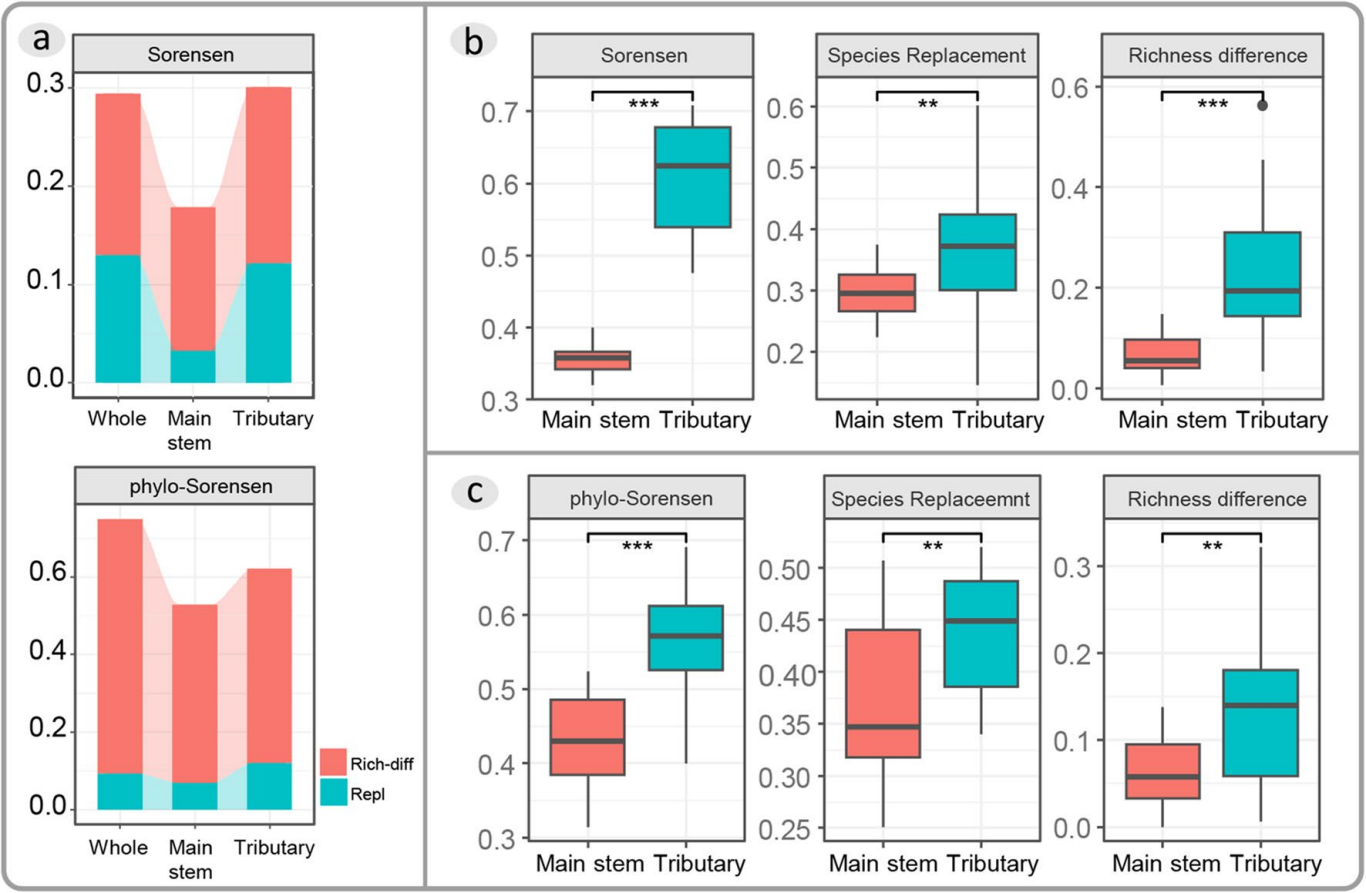
Comparison of taxonomic and phylogenetic Sorensen dissimilarity, as well as components (species replacement and richness difference). **a** Composition of taxonomic beta diversity and phylogenetic beta diversity; **b** Comparison of taxonomic Sorensen dissimilarity between the main stem and tributaries; **c** Comparison of phylogenetic Sorensen dissimilarity between the main stem and tributaries

Phytoplankton assemblies in both tributaries and the main stem were dominated by turnover either in taxonomic or phylogenetic beta diversity (Fig. 3a). The taxonomic Sorensen dissimilarity and phylogenetic Sorensen dissimilarity in tributaries were significantly higher than those in the main stem. Both the species turnover and richness difference of the Sorensen dissimilarity and the phylogenetic Sorensen dissimilarity in in tributaries were notably higher than those in the main stem (Fig. 3b, c).

### Assembly Processes and Source Analysis

**Fig. 4:**
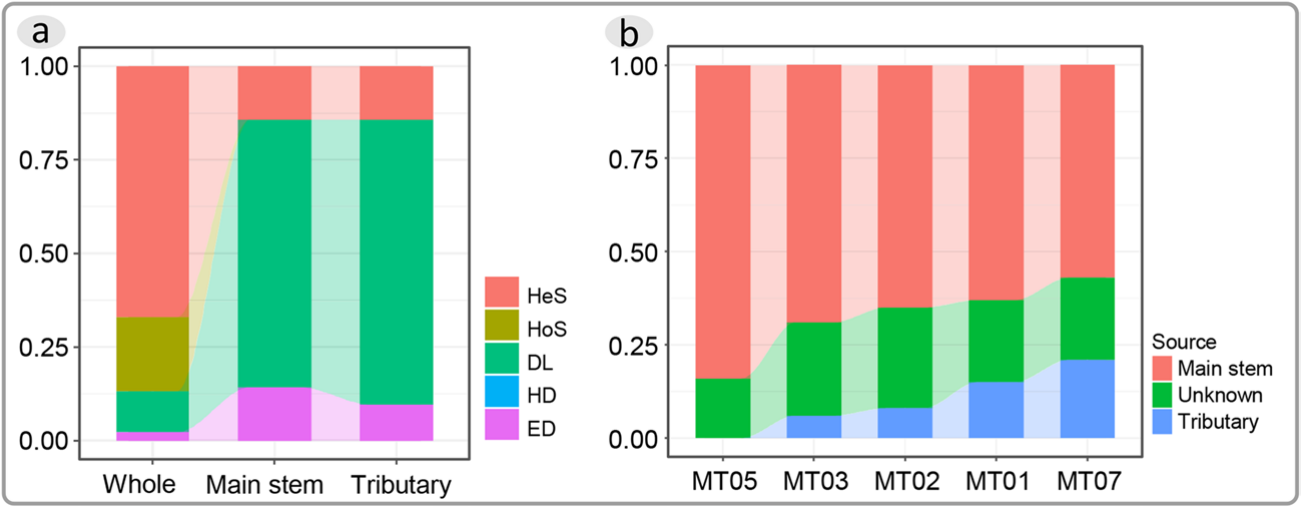
**a** Proportions of different assembly processes in the phytoplankton assemblies of the Medog segment and its main stem and tributaries of the Yarlung Zangbo River. **b** Composition of planktonic algal sources in the assemblies from upstream to downstream at the 5 main stem sampling sites

Overall, the phytoplankton assemblies were influenced by four processes: heterogeneous selection, homogeneous selection, dispersal limitation, and ecological drift. Heterogeneous selection has the highest proportion, with the ration up to 0.63 (Fig. 4a). The phytoplankton assemblies only in the main stem or tributaries are influenced by three processes: heterogeneous selection, ecological drift, and dispersal limitation. The proportion of heterogeneous selection is consistent in both the main stem and tributaries, with a value of 0.14. Dispersal limitation has proportions of 0.72 in the main stem and 0.76 in tributaries. The proportion of ecological drift is 0.14 in the main stem and 0.10 in tributaries (Fig. 4a).

Planktonic algal sources were classified into three parts: main stem, tributary, and unknown. The analysis results for the five main stem sampling points indicated that there were no significant differences in the proportion of unknown sources among these points, ranging from 0.16 to 0.27. The proportion of planktonic algae originating from the upstream main stem point varied from 0.57 to 0.84 (Fig. 4b). With an increasing number of studied tributaries, the proportion of main stem sources showed a decreasing trend. From upstream to downstream, there was a gradual increase in the proportion of planktonic algae originating from the tributaries (Fig. 4b).

### Driving Factors

**Fig. 5:**
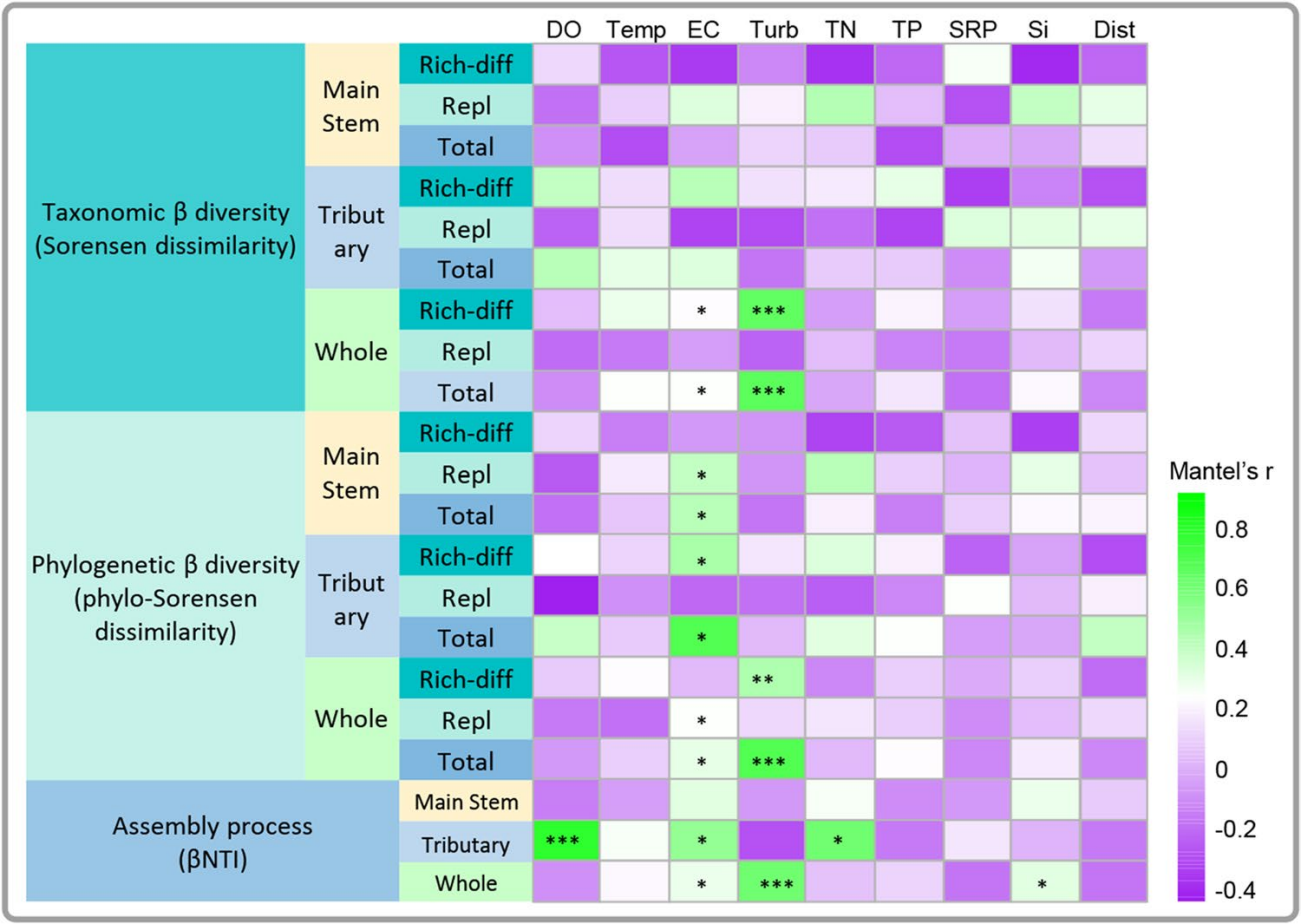
Mantel test analysis of beta diversity (including taxonomic and phylogenetic components), community assembly processes (βNTI), with physicochemical factors, nutrient concentrations, and geographic distance among assemblies. *, **, and *** indicate *p* < 0.05, < 0.01, < 0.001, respectively

The taxonomic beta diversity of planktonic algae in both the main stem and tributaries appears unaffected by most physicochemical factors, except pH. Geographical distances between sampling sites also show no significant correlation (Fig. 5). However, taxonomic Sorensen dissimilarity across all assemblies’ correlates significantly with EC (*r* = 0.2541, *p* = 0.047) and Turb (*r* = 0.6722, *p* = 0.001). This correlation is mainly attributed to the impact of EC (*r* = 0.2322, *p* = 0.05) and Turb (*r* = 0.6605, *p* = 0.001) on richness difference. The phylogenetic Sorensen dissimilarity of both main stem (*r* = 0.4244, *p* = 0.032) and tributaries (*r* = 0.6928, *p* = 0.001) significantly correlates with EC. However, there is a distinction in how EC affects main stem and tributaries assemblies. EC influences species replacement in main stem (*r* = 0.4005, *p* = 0.045), while affecting the richness difference (*r* = 0.4651, *p* = 0.039) in tributaries. Considering both tributaries and the main stem together, phylogenetic Sorensen dissimilarity primarily correlates with EC (*r* = 0.3065, *p* = 0.029) and Turb (*r* = 0.6919, *p* = 0.001), consistent with taxonomic Sorensen dissimilarity results. Notably, EC significantly influences the species replacement (*r* = 0.2513, *p* = 0.013) of overall phylogenetic Sorensen dissimilarity (Fig. 5). The βNTI of main stem assemblies shows no significant correlation with water physicochemical factors, nutrient concentrations, or geographical distances between sampling points. In contrast, the βNTI of tributaries significantly correlates with DO (*r* = 0.7872, *p* = 0.001), EC (*r* = 0.5187, *p* = 0.047), and TN (*r* = 0.6123, *p* = 0.012). Overall, community assembly processes in the main stem and tributaries of the Yarlung Zangbo river in the Medog section are significantly correlated with EC (*r* = 0.2904, *p* = 0.027) and Turb (*r* = 0.6146, *p* = 0.001) (Fig. 5).

## Discussion

### Biomass and Species Distribution Between Main Stem and Tributaries

Despite the extremely low phytoplankton biomass levels in both the main stem and tributaries of the Medog section of the Yarlung Zangbo River, the biomass in tributaries remains significantly higher than that in the main stem. We hypothesize that this phenomenon is primarily driven by Turb. Studies indicate a positive correlation between water turbidity and algal biomass within a certain range, and excessively high turbidity and suspended particulate matter (TSS) can inhibit algal growth [4]. In this study, turbidity in the main stem is relatively high, while most tributaries have extremely low turbidity levels. This allows phytoplankton in tributaries to access more sunlight for photosynthesis, resulting in higher Chla concentrations in tributaries than in main stem. Although tributaries seem more conducive to algal growth from the perspective of Turb, the species richness of phytoplankton in the main stem of the Medog section is significantly higher than that in tributaries. We speculate that this phenomenon is likely due to the unique hydrological characteristics of the Medog section of the Yarlung Zangbo River. The strong water flow in the main stem hinders the dispersal of phytoplankton and their remnants upstream. Consequently, the main stem functions more like a large species pool, collecting phytoplankton and remnants from various upstream tributaries [32]. The use of eDNA methods facilitates the detection of a greater species diversity in the main stem. Source tracing analysis of phytoplankton in the main stem supports this speculation, indicating that including more tributaries increases the proportion of tributary components in the main stem.

### Beta Diversity and Driving Factors

In general, beta diversity is mainly shaped by species turnover (replacement) and differences in species richness (nestedness) between different assemblies 15. With increasing spatial scale and gradients, environmental heterogeneity typically changes, leading to variations in species richness and species turnover between assemblies, further increasing dissimilarity between assemblies [16]. In current study of the Yarlung Zangbo River, turnover significantly contributed to beta diversity either in main stem or tributaries. Taxonomic beta diversity of phytoplankton was not significantly correlated with measured environmental factors in either the main stem or tributaries. However, when considering the entire Yarlung Zangbo system, overall taxonomic beta diversity showed a significant correlation with conductivity and water turbidity. These factors mainly influenced the nestedness process and overall beta diversity. This correlation is likely attributed to notable gradient differences in conductivity and water turbidity between the main stem and tributaries, along with distinct species composition variations among different sampling points [13]. Additionally, tributaries exhibited higher environmental heterogeneity compared to the main stem, resulting in greater community dissimilarity. These findings suggest the potential existence of algae in the Medog section of the Yarlung Zangbo River that are highly specialized to specific habitats (such as conductivity and water turbidity) and may be unique to certain tributaries [20, 21, 32].

Unlike taxonomic beta diversity, phylogenetic beta diversity in both main stem and tributaries were notably correlated with EC in the Medog section of the Yarlung Zangbo River. EC primarily drove the species replacement in the main stem assemblies, while it predominantly influenced the richness difference in tributaries. This implies that differences among tributaries result from conductivity-induced variations in species richness, whereas differences among main stem assemblies mainly from turnover [32]. Significant environmental factors affecting phytoplankton community phylogenetic beta diversity included Turb and EC. Similar to taxonomic beta diversity, Turb mainly influenced the richness difference between main stem and tributary sampling points, while EC impacted the species replacement between these points. This suggests that Turb disrupts species numbers in the Medog section, leading to the loss of certain species due to changes in water turbidity. Conversely, EC and nitrate nitrogen influence species composition, implying a potential competition or selection within the phytoplankton community in response to changes in water conductivity and nitrate nitrogen.

Spatial correlation as an important factor in community ecology is typically taken into account [6]. Normally, phytoplankton assemblies in the main stem should exhibit a significant correlation with hydrological distance when tributary inflow is not considered or inflow is relatively uniform. However, the present results did not support this hypothesis, suggesting that it might be due to the non-uniformity of tributary inflow. The varying number and water volume of tributaries, along with the quantity of phytoplankton (and their remnants) they carry, are challenging to equalize within the same distance. This leads to a lack of a significant correlation between community dissimilarity in the main stem phytoplankton and spatial distance [26]. Additionally, the consistent addition of some components of phytoplankton assemblies from unknown sources in the main stem is also a partial reason for this result.

### Assembly Processes and Mechanisms

The balance between niche-based and stochastic processes in community assembly often changes with hydrological patterns; for instance, plankton assemblies in rivers with high flow rates, such as those in mountain streams, tend to have a higher proportion of stochastic processes [2]. Dispersal limitation can lead to turnover processes between different assemblies, increasing dissimilarity between assemblies [19]. In this study, the assembly processes of different assemblies within the main stem and between tributaries were both dominated by dispersal limitation. This is consistent with the conclusion drawn from beta diversity analysis that turnover between assemblies dominates both in the main stem and between tributaries, indicating that the main reason for community differences between main stem and tributaries is species replacement between different assemblies [33]. This conclusion aligns with the unique habitat characteristics of the Medog section of the Yarlung Zangbo River. The high flow rate restricts bidirectional flow of phytoplankton. Additionally, different tributaries are significantly affected by environmental factors such as EC, DO, and nitrogen nutrients (TN), leading to differences in phytoplankton community composition between them. In contrast, main stem sampling points show similarity both upstream and downstream. However, as more adjacent main stem sampling points include different tributaries, their community similarity decreases. Although the assembly processes within tributaries and main stems are dominated by dispersal limitation, the overall phytoplankton community assembly process in the Medog section of the Yarlung Zangbo River is mainly determined by deterministic processes (heterogeneous selection).

## Conclusion and Outlook

The phytoplankton in the Medog section of the Yarlung Zangbo River exhibits a unique and diverse pattern, especially evident in three aspects: (1) alpha diversity in tributaries is significantly lower than in the main stem; however, biomass in tributaries is significantly higher than main stem; (2) beta diversity and its components in tributaries shows significantly higher than the main stem; (3) the Medog section of the Yarlung Zangbo River is mainly governed by deterministic processes overall, and both main stems and tributaries are dominated by dispersal limitation. The unique pattern of phytoplankton diversity in the Medog section is attributed to the distinctive river habitats, characterized by significant differences in water turbidity, conductivity, and nitrogen nutrients between main stems and tributaries, as well as the short-distance vertical drops and fast water flow in rivers within Medog, hindering the reverse dispersal and exchange of phytoplankton and their remnants in these water bodies.

Global changes, including human activities and climate change, are the primary factors affecting regional ecosystems of Medog. Changes in water flow from climate change in the upstream of the Yarlung Zangbo River and changes in hydrological conditions caused by human activities (such as road and dam construction) are likely to alter the regional river habitat characteristics in the Medog section. This will, in turn, impact aquatic organisms, especially phytoplankton. From the conclusions drawn in this study, continuous long-term monitoring is essential to assess and quantify the impact of climate change and human activities on aquatic organisms, particularly phytoplankton, in the Medog. Protection of the aquatic biodiversity in the downstream Yarlung Zangbo River should primarily focus on tributary waters.

### Supplementary Information

Below is the link to the electronic supplementary material.

Supplementary file1 (DOCX 15 KB)

## Data Availability

The raw data of all samples generated in present work are available in the Sequence Read Archive (SRA, National Center for Biotechnology Information) under the accession number PRJNA1092358.
