# Supplementary material for: Diversity Patterns of Eukaryotic Phytoplankton in the Medog Section of the Yarlung Zangbo River
Source: Microb Ecol. 2024 Apr 15;87(1):59. doi: 10.1007/s00248-024-02371-6 (PMC11018697; doi:10.1007/s00248-024-02371-6)
Supplement: Supplementary file 1 — Supplementary file1 (DOCX 15 KB) [file 248_2024_2371_MOESM1_ESM.docx]

Table S1 the coordinates and environmental variables of each sampling site

| Sites | Lon | Lat | pH | DO (mg/L) | Temp (K) | EC (μs/cm) | Turb (NTU) | TN (mg/L) | TP (mg/L) | SRP (mg/L) | Si (mg/L) | NH_4_ (mg/L) | Vel (m/s) |
| --- | --- | --- | --- | --- | --- | --- | --- | --- | --- | --- | --- | --- | --- |
| MT01 | 95.08867 | 29.20174 | 7.96 | 8.87 | 293.15 | 162.6 | 69.1 | 0.51 | 0.04 | 0.01 | 0.87 | 0.22 | 1.89 |
| MT02 | 95.12962 | 29.22676 | 8.07 | 8.74 | 289.85 | 166.4 | 45.1 | 0.15 | 0.14 | 0.01 | 0.96 | 0.10 | - |
| MT03 | 95.25113 | 29.27919 | 8.27 | 10.52 | 288.95 | 175.6 | 63.9 | 0.31 | 0.07 | 0.04 | 0.92 | 0.37 | 1.38 |
| MT04 | 95.29033 | 29.3280 | 8.16 | 10.51 | 289.55 | 182.5 | 55.1 | 0.50 | 0.16 | 0.08 | 0.93 | 0.32 | - |
| MT05 | 95.40316 | 29.41816 | 7.95 | 11.04 | 288.85 | 183.7 | 73.7 | 0.31 | 0.08 | 0.02 | 0.92 | 0.82 | - |
| MT06 | 95.38052 | 29.66789 | 8.14 | 8.95 | 288.55 | 192.9 | 50.6 | 0.59 | 0.13 | 0.03 | 0.92 | 0.14 | 1.6 |
| MT07 | 95.00329 | 29.18081 | 7.97 | 9.02 | 289.35 | 177.3 | 37.1 | 0.38 | 0.08 | 0.02 | 0.94 | 0.92 | - |
| MT08 | 95.05352 | 29.19022 | 7.76 | 9.29 | 289.55 | 65.1 | 0.78 | 0.35 | 0.02 | 0.01 | 0.90 | 0.07 | - |
| MT09 | 95.08721 | 29.21745 | 7.94 | 8.56 | 291.75 | 119.6 | 12.18 | 0.45 | 0.03 | 0.02 | 1.49 | 0.05 | - |
| MT10 | 95.1681 | 29.30062 | 7.52 | 9.56 | 286.55 | 32.0 | 1.28 | 0.30 | 0.05 | 0.02 | 0.73 | 0.07 | - |
| MT11 | 95.1307 | 29.22542 | 7.24 | 9.43 | 289.35 | 22.8 | 22.5 | 0.21 | 0.15 | 0.02 | 1.84 | 0.29 | 1.79 |
| MT12 | 95.25062 | 29.27491 | 7.47 | 9.88 | 288.05 | 40.6 | 4.04 | 0.11 | 0.04 | 0.04 | 1.68 | 0.07 | - |
| MT13 | 95.28228 | 29.2997 | 7.33 | 9.36 | 289.25 | 27.3 | 3.89 | 0.26 | 0.03 | 0.03 | 1.47 | 0.12 | - |
| MT14 | 95.6357 | 29.4364 | 7.91 | 9.3 | 284.25 | 56.3 | 9.27 | 0.15 | 0.03 | 0.01 | 0.88 | 0.13 | - |
